# Clustering of samples and variables with mixed-type data

**DOI:** 10.1371/journal.pone.0188274

**Published:** 2017-11-28

**Authors:** Manuela Hummel, Dominic Edelmann, Annette Kopp-Schneider

**Affiliations:** Division of Biostatistics, German Cancer Research Center, Heidelberg, Germany; Jiangnan University, CHINA

## Abstract

Analysis of data measured on different scales is a relevant challenge. Biomedical studies often focus on high-throughput datasets of, e.g., quantitative measurements. However, the need for integration of other features possibly measured on different scales, e.g. clinical or cytogenetic factors, becomes increasingly important. The analysis results (e.g. a selection of relevant genes) are then visualized, while adding further information, like clinical factors, on top. However, a more integrative approach is desirable, where all available data are analyzed jointly, and where also in the visualization different data sources are combined in a more natural way. Here we specifically target integrative visualization and present a heatmap-style graphic display. To this end, we develop and explore methods for clustering mixed-type data, with special focus on clustering variables. Clustering of variables does not receive as much attention in the literature as does clustering of samples. We extend the variables clustering methodology by two new approaches, one based on the combination of different association measures and the other on distance correlation. With simulation studies we evaluate and compare different clustering strategies. Applying specific methods for mixed-type data proves to be comparable and in many cases beneficial as compared to standard approaches applied to corresponding quantitative or binarized data. Our two novel approaches for mixed-type variables show similar or better performance than the existing methods *ClustOfVar* and bias-corrected mutual information. Further, in contrast to ClustOfVar, our methods provide dissimilarity matrices, which is an advantage, especially for the purpose of visualization. Real data examples aim to give an impression of various kinds of potential applications for the integrative heatmap and other graphical displays based on dissimilarity matrices. We demonstrate that the presented integrative heatmap provides more information than common data displays about the relationship among variables and samples. The described clustering and visualization methods are implemented in our R package *CluMix* available from https://cran.r-project.org/web/packages/CluMix.

## Introduction

In real data situations various factors of interest are measured on different scales, e.g. quantitative gene expression values and categorical clinical features like gender, disease stage etc. In many cases high-dimensional data are analyzed first, and further patient characteristics are only added “informatively” to presented results. This is, however, unsatisfactory from a systems biology point of view. There is a growing number of integrative approaches that combine different data sources from the beginning of the analysis, instead of post-hoc combining the results derived in separate steps, e.g. integrative clustering [1], multiple factor analysis with mixed data [2], or Bayesian approaches [3, 4]. In this work we follow a similar direction, however, our focus lies more in the visualization, driven by the idea to show the “complete picture” in a heatmap-style presentation. Biclustering is a common approach to detect structures among samples and variables simultaneously, which means essentially to find “blocks” within heatmaps. To the author’s knowledge, however, biclustering methods either apply to quantitative [5] or, in the field of pattern mining, to categorical data [6], but not to a mix of different data types. The R package *caOmicsV* [7] provides a heatmap display for multiple “omics” and phenotypical data. However, the different datasets have to be provided at gene level, for instance DNA methylation, mutations or DNA copynumber variations have to be specified per gene, which is not straight forward. Further, no clustering is applied to samples or features that would allow to discover structures in the data. The integrative clustering approach [1] also provides heatmaps, while the purpose is to find groupings amongst samples, using information from different types of high-dimensional datasets simultaneously. Structures among variables are only displayed within the different datasets. In contrast, we also want to explore similarities between all variables in one unified presentation and further include low-dimensional characteristics, which can be used for explorative analysis and hypothesis generation. For example, one might wish to find subgroups of patients based on all relevant parameters, and simultaneously explore relationships between those parameters. Another example is the inspection of associations between variables in the process of statistical modelling, prior to deciding about their inclusion or exclusion from a regression model. Finally, integrative illustration plays an important role in presenting results, showing e.g. prognostic factors of different types and their relationship between each other and to the outcome of interest.

In order to create a heatmap for variables measured on different scales, special similarity measures are necessary defining i) distances between samples (e.g. patients) based on features of different types, and ii) distances between the different variables. For clustering samples using mixed-type variables, we choose to use Gower’s similarity coefficient [8]. For clustering variables of different types, we propose two new strategies: 1) The *CluMix-ama* (*a*ssociation *m*easures *a*pproach) method consists in combination of different similarity measures. A novel strategy based on category reordering is suggested for measuring the association between a multi-categorical and any other type of variable. 2) The *CluMix-dcor* (*d*istance *cor*relation) approach is based on a novel similarity measure, which is derived using the concept of generalized distance correlations [9]. Instead of always using the Euclidean distance as in the original definition of the distance correlation [10, 11], we apply distances corresponding to the respective type of variable. In particular, we will use the Euclidean distance for ordered and quantitative variables and the discrete distance for nominal variables.

**Fig 1:**
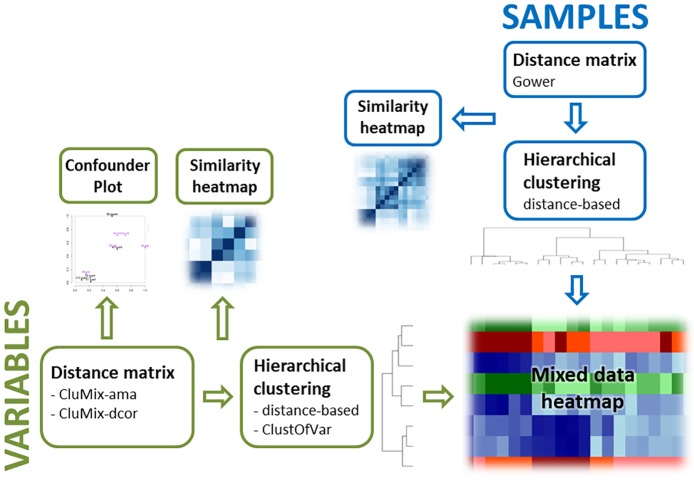
Functionality of the CluMix R package. Distance matrices are derived separately for samples and variables. They build the basis for hierarchical clustering and integrative visualization of mixed data.

Both methods are compared to the *ClustOfVar* approach [12] and clustering based on bias-corrected mutual information (*BCMI*) [13] by simulation studies. Using hierarchical clustering for mixed data, standard heatmaps as for continuous values can be drawn, with the difference that separate color schemes illustrate the differing sources of information. On the basis of the mixed data similarity matrices further simple plots can be constructed that show relationships between variables. The utility of the visualization methods is illustrated with a real data example. The mixed data clustering and visualization tools are implemented in our R package *CluMix*. Fig 1 gives an overview over the functionalities of the package.

## Methods

Table 1 summarizes the most important symbols used in the Methods section to facilitate reading.

**Table 1:** Overview over most important symbols.

*n*	number of samples
*p*	number of variables
*K*	number of categories for nominal or ordinal variables
*S* / *D*	similarity / distance matrix
*x*_*ik*_	value of sample *i* for variable *k*
*s*_*k*_(*x*_*ik*_, *x*_*jk*_) / *d*_*k*_(*x*_*ik*_, *x*_*jk*_)	similarity / distance between samples *i* and *j* based on variable *k*
*s*(*x*_*i*_, *x*_*j*_) / *d*(*x*_*i*_, *x*_*j*_)	similarity / distance between samples *i* and *j* based on all variables
*s*_*kl*_ / *d*_*kl*_	similarity / distance between variables *k* and *l*
*ρ*	correlation coefficient
${\tilde{ℛ}}_{kl}$	generalized distance correlation between variables *k* and *l*

### Clustering samples

We want to cluster samples (e.g. patients) based on properties that can be measured on different scales, i.e. quantitative, ordinal, categorical or binary variables. There is plenty of literature on clustering samples, even for mixed numerical and categorical data, see Table 2 for an overview of the considered methods.

**Table 2:** Methods for clustering or defining distances between samples with mixed data. The columns indicated whether hierarchical clustering is suitable (in contrast to partitioning), whether distance matrices can be retrieved, whether the funcionality is available in R (to the authors’ knowledge) and whether ordinal variables are treated in a special way. Only clustering based on Gower’s similarity coefficient is applied throughout the manuscript.

clustering / distance method	hierarchical	distance matrix	implemented in R	ordinal variables
latent class clustering [14]			✓	✓
k-prototypes [15]			✓	
fuzzy clustering [16]				
Mahalanobis-type distance [17]	✓	✓		
Value difference Metric [18]	✓	✓		
Gower’s similarity coefficient [8]	✓	✓	✓	✓

Most methods, like latent class clustering [14], k-prototypes clustering [15], fuzzy clustering [16] and others [19], aim in partitioning the data into a fixed number of clusters, which is, especially for large datasets, computationally more efficient than hierarchical clustering, where the complete dissimilarity matrix is required. Having a mixed-data heatmap in mind, however, we prefer hierarchical clustering schemes based on dissimilarity matrices, where no fixed number of clusters has to be chosen a priori. In the field of machine learning several approaches exist for evaluating distances between samples using mixed data [17, 18]. However, those approaches are rather complex, and are not tailored for ordinal variables. Instead, we choose the general similarity coefficient proposed by Gower [8] for defining distances between samples. Similarity between samples *i* and *j* with values *x*_*i*_ and *x*_*j*_, *i*, *j* = 1,…,*n*, based on *p* variables, is defined as

$$\begin{array}{c} s\left(x_{i},x_{j}\right)=\sum_{k=1}^{p}s_{k}\left(x_{ik},x_{jk}\right)\delta_{k}\left(x_{ik},x_{jk}\right)w_{k}/\sum_{k=1}^{p}\delta_{k}\left(x_{ik},x_{jk}\right)w_{k} \end{array}$$

where *δ*_*k*_(*x*_*ik*_, *x*_*jk*_) indicates whether a comparison of *i* and *j* is possible on variable *k*, *k* = 1,…,*p*, i.e. *δ*_*k*_(*x*_*ik*_, *x*_*jk*_) = 0 if *i* and/or *j* have a missing value for *k*, and *δ*_*k*_(*x*_*ik*_, *x*_*jk*_) = 1 otherwise. Optional weights *w*_*k*_ can be specified in order to raise importance of certain variables that a priori are considered more relevant. If no such preferences exist, *w*_*k*_ is set to 1 for all *k* = 1,…,*p*. The score *s*_*k*_(*x*_*ik*_, *x*_*jk*_) captures the similarity between samples *i* and *j* w.r.t. variable *k*. In short, the score is defined for

- *qualitative* variables: $s_{k}(x_{ik},x_{jk})=\left\{ \begin{array}{cc} 1, & i\;\text{and}\;j\;\text{agree}\;\text{in}\;k\\ 0, & i\;\text{and}\;j\;\text{differ}\;\text{in}\;k \end{array}\right.$
- *quantitative* variables: *s*_*k*_(*x*_*ik*_, *x*_*jk*_) = 1 − |*x*_*ik*_ − *x*_*jk*_|/*R*_*k*_,
  where *R*_*k*_ is the observed range of variable *k*.

With the extension of Podani [20] it is possible to also incorporate ordering information of variables on ordinal scale

- *ordinal* variables: $s_{k}(x_{ik},x_{jk})=1-\frac{\left|r_{k}(x_{ik})-r_{k}(x_{jk})\right|}{max_{m}\{r_{k}(x_{mk})\}-min_{m}\{r_{k}(x_{mk})\}}$, where *r*_*k*_(*x*_*mk*_) is the rank of value *x*_*mk*_ of sample *m* within all observations for variable *k*.

The method is implemented in the R package *FD* [21].

From the similarity values *s*(*x*_*i*_, *x*_*j*_) we calculate distances *d*(*x*_*i*_, *x*_*j*_) = 1 − *s*(*x*_*i*_, *x*_*j*_). Once a suitable distance matrix is derived, all standard clustering algorithms starting from pairwise dissimilarities can be applied for exploring structures in the data. For visualization purposes we find hierarchical clustering most suitable. In our analyses we use Ward’s method [22] for the calculation of between-cluster distances, but any other linkage method is possible as well. Also partitional clustering approaches can be applied, for example Partitioning Around Medoids (PAM), a more robust and flexible version of the classical k-means algorithm, where a dissimilarity matrix can be chosen by the user [23].

### Clustering variables

Besides clustering samples, we mainly aim in defining similarities between the variables themselves, in order to be able to visualize simultaneously relationships between samples and variables, as common in standard heatmaps. Approaches using mutual information [24] or factor analysis for mixed data [2] could be used to assess associations between features. But those methods on the one hand are quite complex, and on the other hand it is not clear whether the derived variable similarities reflect associations in the spirit of a correlation, which we are most interested in. Nevertheless, we consider the recent bias-corrected mutual information (*BCMI*) [13], that is implemented in R package *mpmi*, for clustering variables by defining distances as 1 − *BCMI*. We further evaluate the non-similarity based approach *ClustOfVar* [12]. Here we propose two alternatives, where the first is a combination of single association measures for different pairs of data types. We call this strategy the *CluMix-ama* approach. The second approach makes use of distance correlation for calculating distances between variables, and is called in the following the *CluMix-dcor* approach. See Table 3 for an overview of the considered methods.

**Table 3:** Like Table 2, but for methods for clustering or defining distances between *variables* of mixed types. The last four methods in the table are applied and compared throughout the manuscript.

clustering / distance method	hierarchical	distance matrix	implemented in R	ordinal variables
factor analysis for mixed data [2]	✓	✓	✓	
mutual information [24]	✓	✓	✓	
BCMI [13]	✓	✓	✓	
ClustOfVar [12]	✓	(✓)	✓	
CluMix-ama	✓	✓	✓	✓
CluMix-dcor	✓	✓	✓	✓

#### The CluMix-ama approach

We start with the choice of suitable association measures for different data type comparisons. The selected similarity coefficients should use as much information as possible (e.g. categorization of quantitative variables shall be avoided), but be as robust as possible (e.g. against outliers and non-linearity). Our decisions on specific measures were based on literature research [25, 26] and a small simulation study. In some cases where no similarity coefficient is readily available, e.g. for measuring the relationship between a continuous and a categorical feature with more than two categories, extensions of existing measures are suggested. The following coefficients are used for measuring the similarity between variables *k* and *l* with respective scales

- *quantitative versus quantitative/ordinal*: absolute Spearman correlation coefficient (i.e. the Pearson correlation on ranks *r*_*k*_ and *r*_*l*_ of values *x*_*k*_ and *x*_*l*_ of variables *k* and *l*)
  *s*_*kl*_ = |*ρ*_*Spearman*_(*x*_*k*_, *x*_*l*_)| = |*ρ*_*Pearson*_(*r*_*k*_, *r*_*l*_)|
- *ordinal versus ordinal* and *quantitative/ordinal versus binary*: absolute Goodman and Kruskal’s *γ* coefficient [27]
  *s*_*kl*_ = |(*n*_*c*_ − *n*_*d*_)/(*n*_*c*_ + *n*_*d*_)|
  where *n*_*c*_ and *n*_*d*_ are the numbers of concordant and discordant pairs of observations w.r.t. *k* and *l*.
- *quantitative/ordinal versus nominal*: No suitable coefficient of association between a rank-order variable and a nominal factor with more than two categories without any natural ordering could be found in the literature. To evaluate the association between those kinds of variables, we apply the idea that a nominal variable could be considered as ordinal, if we would only know the “correct” ordering. As an example, consider a nominal factor *X* with categories *A*, *B* and *C*, and a quantitative variable *Y* that is associated with *X* in the way that it shows similar values in samples with levels *A* and *C*, but elevated values in samples with level *B*. Hence, we could measure this association by calculating the Spearman correlation coefficient (if *Y* is quantitative) or Goodman and Kruskal’s *γ* (if *Y* is ordinal) for *X*′ and *Y*, where *X*′ is *X* transformed to an ordered factor with levels *A* < *C* < *B*. To define the “correct” ordering of categories of *X* with respect to variable *Y*, we consider the average ranks of *Y* values within the respective categories of *X*. Since in the case of no real relationship between *X* and *Y* this strategy would yield too optimistic estimations of association, we first perform a Kruskal-Wallis test to screen for any difference in means of *Y* within the categories of *X*. Only if the test result is significant (*p* < 0.05) we go on with the reordering as described. In the opposite case, we calculate the Spearman correlation or respectively Goodman and Kruskal’s *γ* using the original *X*, which would represent a “random” ordering of categories and should lead to a coefficient close to 0.
- *nominal/binary versus nominal/binary*: There are measures of association of cross-table data, like e.g. Pearson’s contingency coefficient or Cramer’s V coefficient. However, in simulations we found more suitable a strategy similar to the one described above, where an ordering is “imposed” into the categorical variables. This idea was already described e.g. in [28] and [29], and is also used in correspondence analysis [30]. The “correct” ordering of categories is achieved by “diagonalizing” the cross-table between the two factors, with the goal to obtain in the diagonal large frequencies. Goodman and Kruskal’s *γ* coefficient is then calculated for the reordered cross-table. Since again in the case of no association this strategy would lead to over-optimistic results, a chi-square pre-test of association is performed prior to optimizing the cross-table. In case of a non-significant test result (*p* > 0.05), the *γ* coefficient is calculated for the original contingency table.

Even though for clustering not absolutely necessary, it would be beneficial for the chosen distances to have metric properties, which implies in especially that the triangle inequality holds for each triplet of distances. Gower [31] shows that if a similarity matrix *S* = (*s*_*kl*_) is positive semi-definite (p.s.d.), then the distance matrix $D=d_{kl}=\sqrt{1-s_{kl}}$ is Euclidean, which of course implies that it is metric. Combining the proposed measures of association to a similarity matrix *S*, it is easy to find an example where *S* is not p.s.d. In order to still fulfill the triangle inequality and hence all the distances to be comparable, we computationally find a similarity matrix *S*′ that is p.s.d. and is “closest” to the original matrix *S* in the sense that the weighted Frobenius norm of the difference of the two matrices is minimized [32]. This method is implemented in the function *nearPD* in R package *Matrix* [33]. By Gower’s theorem then follows that the distance matrix *D* composed by inter-variable distances $d_{kl}=\sqrt{1-s_{kl}}$ is Euclidean. Based on the distance matrix *D*, grouping of variables can be conducted again by standard hierarchical or partitional clustering.

#### The CluMix-dcor approach

The distance covariance and the distance correlation are novel measures of dependence, which were originally proposed for the univariate setting by Feuerverger [34] and later extended to multivariate observations by Székely [10, 11]. Since the appearance of [10, 11], considerable interest in statistical applications of the distance correlation coefficient has arisen. Notably, distance correlation has been used for inferring gene regulatory networks [35], testing associations between the copy number variations of different genes [36] and assessing associations of familial relationships, lifestyle factors and mortatility [37].

The distance correlation coefficient ℛ(X,Y) is a measure of dependence between a *p*-dimensional random vector *X* and a *q*-dimensional random vector *Y*, where *p* and *q* are arbitrary. The distance correlation is always positive with 0≤ℛ(X,Y)≤1 and it equals 0 if and only if the vectors *X* and *Y* are independent. This property implies that the distance correlation can detect any dependence between *X* and *Y*. Recently, Lyons [9] generalized this concept to metric spaces. Given any two metric spaces (i.e. appropriate sets on which proper distances are defined), one can derive a generalized distance correlation between random variables on these two different sets. When the metric spaces satisfy an additional property, called strongly negative type, we even retain the property that this generalized distance correlation is 0 if and only if the two random variables are independent. By reducing the values of these random variables to the distances, this approach allows to measure dependence between two random variables on completely different sets. In this article, we will restrict ourselves to measuring dependence between two variables, where any of those two variables may be either quantitative, ordinal or nominal. In particular, similar to what was used for defining distances between samples, for *k* ∈ {1,…,*p*}, we let

- $d_{k}(x_{ik},x_{jk})=\left\{ \begin{array}{cc} 0, & x_{ik}=x_{jk}\\ 1, & \text{else}, \end{array}\right.$ if the *k*-th variable is *nominal*,
- *d*_*k*_(*x*_*ik*_, *x*_*jk*_) = |*x*_*ik*_ − *x*_*jk*_| if the *k*-th variables is *quantitative*,
- *d*_*k*_(*x*_*ik*_, *x*_*jk*_) = |*r*_*k*_(*x*_*ik*_) − *r*_*k*_(*x*_*jk*_)| if the *k*-th variables is *ordinal*, where *r*_*k*_(*x*_*ik*_) and *r*_*k*_(*x*_*jk*_) are the ranks of values *x*_*ik*_ and *x*_*ik*_ within all observations for variable *k*.

We define the centered distance matrices for the *k*-th and *l*-th variable, respectively, as

$$\begin{array}{cc} A_{ij} & =d_{k}\left(x_{ik},x_{jk}\right)-\frac{1}{n}\sum_{i=1}^{n}d_{k}\left(x_{ik},x_{jk}\right)-\frac{1}{n}\sum_{j=1}^{n}d_{k}\left(x_{ik},x_{jk}\right)+\frac{1}{n^{2}}\sum_{i,j=1}^{n}d_{k}\left(x_{ik},x_{jk}\right),\\ B_{ij} & =d_{l}\left(x_{il},x_{jl}\right)-\frac{1}{n}\sum_{i=1}^{n}d_{l}\left(x_{il},x_{jl}\right)-\frac{1}{n}\sum_{j=1}^{n}d_{l}\left(x_{il},x_{jl}\right)+\frac{1}{n^{2}}\sum_{i,j=1}^{n}d_{l}\left(x_{il},x_{jl}\right). \end{array}$$

Now, by multiplying these matrices containing *distances between the samples*, we obtain a *similarity measure between the variables*. In particular a sample version of the generalized distance covariance [9, pp. 3287] between the *k*-th variable and the *l*-th variable is defined as the nonnegative squareroot of

$$\begin{array}{c} {\hat{U}}_{kl}=\frac{1}{n^{2}}\sum_{i,j=1}^{n}A_{ij}B_{ij}. \end{array}$$

This sample measure is known to be severely biased [38]. For better performance, we will use a bias-corrected version of ${\hat{U}}_{kl}$ in the fashion of [38]:

$$\begin{array}{c} {\tilde{U}}_{kl}=\frac{n-3}{n}(\sum_{i,j=1}^{n}A_{ij}^{*}B_{ij}^{*}-\frac{n}{n-2}\sum_{i=1}^{n}A_{ii}^{*}B_{ii}^{*}), \end{array}$$ (1)

where the modified distance matrix $A_{ij}^{*}$ is

$$\begin{array}{c} A_{ij}^{*}=\{\begin{array}{cc} \frac{n}{n-1}(A_{ij}-\frac{d_{k}\left(x_{ik},x_{jk}\right)}{n}) & \text{for}\;i\neq j,\\ \frac{n}{n-1}(\frac{1}{n}\sum_{j=1}^{n}d_{k}\left(x_{ik},x_{jk}\right)-\frac{1}{n^{2}}\sum_{i,j=1}^{n}d_{k}\left(x_{ik},x_{jk}\right)) & \text{for}\;i=j, \end{array} \end{array}$$

and $B_{ij}^{*}$ is defined analogously. Different from ${\tilde{U}}_{kl}$, ${\tilde{U}}_{kl}$ may take negative values. By normalizing ${\tilde{U}}_{kl}$, we obtain a generalized distance correlation:

$$\begin{array}{c} {\tilde{R}}_{kl}=\mathrm{sign}\left({\tilde{U}}_{kl}\right)\;\sqrt{\frac{|{\tilde{U}}_{kl}|}{\sqrt{{\tilde{U}}_{kk}\;{\tilde{U}}_{ll}}}}. \end{array}$$ (2)

Here ${\tilde{U}}_{kk}$ and ${\tilde{U}}_{ll}$ refer to the squared generalized distance variance of the *k*-th and the *l*-th variable, respectively (these are calculated by plugging in the distances from one and the same variable into Eq (1)). As long as only quantitative variables are involved, ${\tilde{ℛ}}_{kl}$ reduces to a bias-corrected version of the standard distance correlation as defined by Székely [10, 11, 38].

Using the fact that both the Euclidean distance and the discrete distance on a countable set are of strongly negative type, we can deduce that [9, Theorem 3.11] ${\tilde{ℛ}}_{kl}$ converges almost surely to 0 if and only if the *k*-th and the *l*-th variable are independent. Hence, the dissimilarity measure $d_{kl}\;≔\;1-{\tilde{ℛ}}_{kl}$ is a meaningful measure for clustering mixed variables.

One can deduce from Eq (2) that the distance correlation is scale-invariant. Hence, for calculating the distance correlation coefficient ${\tilde{ℛ}}_{kl}$, it does not make a difference if we replace the metric used for quantitative variables by *d*_*k*_(*x*_*ik*_, *x*_*jk*_) = |*x*_*ik*_ − *x*_*jk*_|/*R*_*k*_ and the metric used for ordinal variables by $\frac{\left|r_{k}(x_{ik})-r_{k}(x_{jk})\right|}{max_{m}\{r_{k}(x_{mk})\}-min_{m}\{r_{k}(x_{mk})\}}$, where *R*_*k*_ is the range of the *k*-th variable and *r*_*k*_(*x*_*ik*_) and *r*_*k*_(*x*_*jk*_) are the ranks of values *x*_*ik*_ and *x*_*jk*_ for variable *k*. These distances are exactly the summands showing up in the Gower’s distance, which we use for clustering samples. This analogy can lead to a saving of computation time when simultaneously clustering samples and variables.

#### Non-similarity-based clustering

The ClustOfVar method [12], implemented in the R package with same name, is based on principal component analysis and was specifically designed for clustering variables. The authors provide a partitioning algorithm that is based on between-variable similarities. They use squared canonical correlation for this purpose. In a basic simulation study we observed, however, that, if similarity is to be measured in the spirit of a correlation, those similiarties often underestimate truly underlying strong relationships. The authors themselves state that their alternative hierarchical clustering method usually performs better. This method does not work with a pre-specified complete distance matrix plugged into the usual hierarchical clustering algorithm, but rather decides on agglomerating sub-clusters to clusters based on the adequacy between the variables within a cluster and a synthetic quantitative variable that is “representative” for the cluster. The synthetic variables are calculated by principal components analysis for mixed data (PCAMIX), and a measure for the mentioned adequacy is the respective first eigenvalue of the PCAMIX decomposition.

## Results: Simulation studies

### Validation of similarity measures between variables

In order to evaluate the between-variable similarities calculated for the CluMix-ama approach, datasets with different kinds of variables were simulated in the following way. First, random normally distributed variables were generated with a specified Pearson correlation between each other. Categorical factors were then created in “perfect agreement” with the quantitative variables. For example, to create a binary variable from a continuous variable *X* that should have the same amount of association as *X* itself to another continuous variable *Y*, *X* was categorized by a median cut. Similarly, to get a factor with four levels, *X* was cut at its quartiles, and so on. In this way datasets with known relationships between the different variables were created. For the selection of suitable similarity coefficients for the CluMix-ama approach, for each pair of different variable types several association measures were tested, see S1 Fig. The coefficients suggested in the Methods section (indicated in bold in S1 Fig) show the least “over-optimism” in the case of no relation and capture best strong relationships.

**Fig 2:**
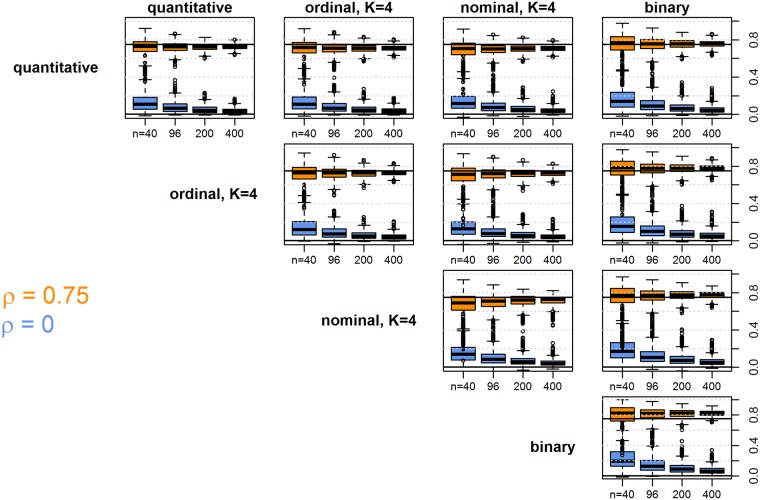
Boxplots of variable similarities for 1000 simulated datasets in different settings. Variables were simulated with an underlying (Pearson) correlation of *ρ* = 0 (blue boxes) and *ρ* = 0.75 (orange boxes), indicated by horizontal lines. The quantitative variables were categorized in perfect agreement, such that the association should be the same between respective variables. The different panels show similarity values calculated for different combinations of data types, as indicated on top and to the left of each column/row of plots. Within each panel, results for different sample sizes (*n* = 40, 96, 200, 400; boxplots from left to right) are shown.

Next, we explored in more detail how well the imposed (Pearson) correlations were captured by the association measures applied to different combinations of data types. We simulated 1000 datasets for every combination of several imposed correlations between variables (*ρ* = 0, 0.25, 0.5, 0.75, 0.95) and sample sizes (*n* = 40, 96, 200, 400—we chose sample sizes divisible by 8, such that the variable categorization into *K* = 8 classes was perfectly balanced). The results are shown in Fig 2, exemplary for correlation values of *ρ* = 0 and *ρ* = 0.75. The larger the sample size, the closer we get to the imposed values of correlation. For small sample sizes, we observe some over-estimation of the truly non-existing relationship (*ρ* = 0, upper panel) for the newly proposed strategy of category reordering to assess relationships between nominal factors and variables of any other type. But, thanks to the pre-test of association the results still seem acceptable (for instance, over-estimation is not much more severe than e.g. for Spearman correlation when relating two quantitative variables). Standard measures for comparing nominal variables (e.g. Cramer’s V) would actually be more over-optimistic in the case of no real association. Further, for larger values of *ρ* those measures sometimes strongly under-estimate the association (see S1 Fig). Strong associations (lower panel) are captured well in general by the proposed measures. Results for ordinal and nominal variables with *K* = 8 instead of four categories are very similar (data not shown). For the CluMix-dcor approach, we do not provide a similar plot. This is for the reason that, even for two quantitative variables, the Pearson correlation and the distance correlation between two variables measure two different quantities and can in general not be converted into each other. We note that for the bivariate normal, the distance correlation is always smaller than or equal to the Pearson correlation [10, Theorem 7], the same holds for many other parametric distributions [39].

### Variables clustering validation

In another simulation study, we explored whether clustering of variables by the suggested methods yield the expected results when the true classifications are known. Datasets with varying sample sizes (*n* = 25, 50, 100) and numbers of variables (*p* = 50, 100, 200) were simulated. Two equally sized groups of variables were defined by separately drawing from multivariate normal distributions with specified intra-group covariance structure. Variables inside a group had pairwise correlations decreasing from varying maximal correlation (*ρ* = 0.25, 0.5, 0.75) to 0. Optionally, some noise was introduced by setting 0%, 20%, 40% of the inter-group correlations to a value of 0.5 instead of 0.

The resulting continuous datasets were clustered using the standard Euclidean distance, as a reference. As a second reference, completely binarized data (cutting values at the median for each variable) were clustered using the simple matching coefficient distance. Subsequently, a certain fraction of variables (10%, 25%, 50%, 75%, 100%) was categorized in the same way as in the previous simulation studies, thus retaining the same underlying true sample and variable classifications. For the categorization, it was randomly decided how many categories (*K* = 2,…,8) a new variable should have, and whether it should be ordinal or nominal. The exclusively quantitative data and its partly or completely categorized variants were clustered with the presented approaches for clustering mixed-type data, namely the CluMix-ama, CluMix-dcor, ClustOfVar and BCMI approaches. Hierarchical clustering with Ward’s agglomeration method was applied and resulting dendrograms were cut in order to detect the two classes of variables. For each setting, simulations were repeated 100 times. Misclassification rates (*MCR* = (*a*_12_ + *a*_21_)/(*a*_11_ + *a*_12_ + *a*_21_ + *a*_22_), with entries *a*_*ij*_ in classification tables) were calculated for evaluation and comparison of the different clustering strategies and simulation settings.

**Fig 3:**
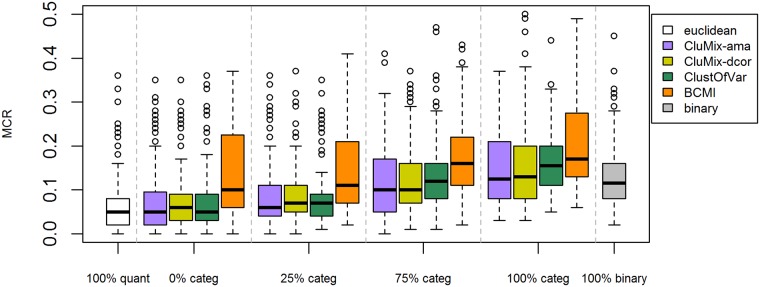
Misclassification rates from clustering variables of 100 simulated datasets each, using the CluMix-ama, CluMix-dcor, ClustOfVar and BCMI approach. Datasets were simulated as described in the Methods section for evaluating clustering of variables. This plot shows results of the simulation setting with 50 samples, 100 variables, within-group correlation of 0.5, and 20% of between-group correlations of 0.5 instead of 0. MCRs (y-axis) were calculated based on clustering with Euclidean distances for the purely quantitative datasets (white), with the three approaches for mixed data (purple: CluMix-ama, yellow: mCluMix-dcor, green: ClustOfVar, orange: BCMI) for datasets with varying amounts of categorical variables (0%, 25%, 75%, 100% from left to right), and with simple matching coefficient for completely binarized data (grey).

**Fig 4:**
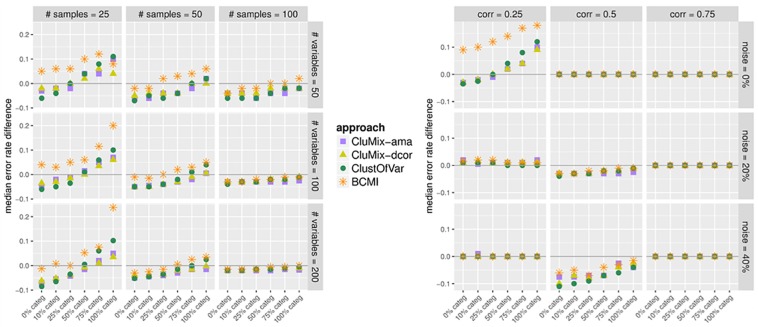
Median difference in error rates between each mixed-data method and binary clustering. For all simulation settings, misclassification rates from clustering binarized datasets by simple matching cofficient as a reference were subtracted from corresponding MCRs when using each of the three mixed-data variables clustering approaches. Shown are the medians of those differences (purple square: CluMix-ama, yellow triangle: CluMix-dcor, green circle: ClustOfVar, orange star: BCMI). In the left panel, different sample sizes (*n* = 25, 50, 100; panel columns) and numbers of variables (*p* = 50, 100, 200; panel rows) were considered, while keeping within-group correlation fixed at 0.5 and fraction of non-zero between-group correlations (with value 0.5) at 20%. In the right panel, settings varied w.r.t. within-group correlations (corr = 0.25, 0.5, 0.75; panel columns) and fraction of between-group correlations with value 0.5 instead of 0 (noise = 0%, 20%, 40%; panel rows), while keeping numbers of samples and variables fixed at 100, respectively. Datasets were simulated with varying amounts of categorical variables (0%–100%; from left to right within each sub-figure).

The misclassification rates for all simulation settings are shown in S2 and S3 Figs. An example is given in Fig 3 for the setting with 50 samples, 100 variables, within-group correlation of 0.5, and 20% of between-group correlations of 0.5 instead of 0. Our two new approaches for mixed-type data and ClustOfVar yield very similar results in terms of recovering the true underlying grouping of variables. For completely quantitative data, those mixed-data clustering methods perform nearly as good as Euclidean clustering. However, the purpose and strength of those methods is of course clustering data including also categorical variables. For such datasets, comparison with clustering completely binarized data by standard methods is more useful. From Fig 3 can be seen that the first three mixed-data approaches generally outperform binary clustering. Only for datasets with exclusively categorical variables dichotomization seems more appropriate. Clustering based on bias-corrected mutual information in this situation performs worse than the other mixed-data approaches and, apart from datasets with few categorical variables, also worse than clustering binary data. S2 and S3 Figs suggest that mutual information works better with larger sample sizes and rather strong correlations among variables. For a better comparison of the methods over all simulation settings, we calculated median differences in MCRs between each method and clustering binarized data, see Fig 4. Median differences below the zero line indicate better performance for the respective mixed-data approach as compared to binary clustering. Again we see that the first three studied methods perform very similarly, while BCMI performs worse, except for large samples sizes and strong correlations. For larger sample sizes (left panel), all four mixed-data approaches outperform binary clustering. For small to moderate sample sizes we observe this benefit only if the fraction of non-quantitative variables does not exceed around 75%. In these situations the CluMix-ama and the CluMix-dcor approach usually yield somewhat better results than ClustOfVar. Also for low within-cluster correlation and no between-cluster correlation (right panel), there is a slight advantage of the two new clustering approaches in case of larger fractions of categorical variables in the data. However, in this situation again dichotomization seems the best option. If there is some amount of between-cluster correlation (“noise”), all methods work equally bad (compare S3 Fig). This is not too surprising, since between-cluster correlation of 0.5 was chosen for 20% or 40% of the variables, which is hence larger than the within-cluster correlation. For moderate within-cluster correlation, however, the mixed-data approaches outperform binary clustering in the presence of noise. When within-cluster correlation is large, all methods work perfectly.

## Results: Real data examples

### Mixed-data versus standard approaches on real data

We wanted to further explore the potential benefit of applying specific mixed-data clustering methods in real situations as compared to standard approaches. For this general purpose we studied clustering of samples, since there are real world examples with a given class membership for samples, whereas a “true” grouping of variables is barely ever known. In three datasets with variables of different types we first pre-selected quantitative variables that are associated with the respective binary outcome of interest. Then we clustered the samples in order to recover the two classes by i) Euclidean distances using only the quantitative variables in the dataset, ii) simple matching distance after dichotomization of the data, iii) the mixed-data approaches latent class, k-prototype and Gower. The performance of the methods is compared by balanced error rates (*BER* = 0.5 ⋅ (*a*_12_/(*a*_11_ + *a*_12_) + *a*_21_/(*a*_21_ + *a*_22_)), where *a*_*ij*_ are the entries of the classification confusion matrix), which is more suitable than the misclassification rate in case of unequal class sizes. The three example datasets are:

**Breast cancer treatment response**: This breast cancer dataset [40] including 133 patients was used for the development of a gene expression-based classifier for pre-operative treatment response. For our analysis, we select the 10 most differentially expressed genes between ‘pathologic complete response’ (34 patients) and ‘residual disease’ (99 patients), and the categorical variables estrogen receptor status, progesterone receptor status, tumor grade and molecular subclass.
**Breast cancer survival**: From the dataset of the Netherlands Cancer Institute for prediction of metastasis-free survival in 144 lymph node positive breast cancer patients [41] we use the landmark of 6-year survival as binary outcome for our analysis (89 patients did and 35 did not survive 6 years; 20 patients censored before reaching 6 years were removed from the analysis). From the published 70 gene signature we select the 10 most differentially expressed genes and categorical variables estrogen receptor status, tumor grade and age class.
**Chemical manufacturing process**: This dataset of 58 measurements on 176 samples is available in the R package *AppliedPredictiveModeling* [42] and contains information about a chemical manufacturing process, in which the goal is to understand the relationship between the process and the resulting final product yield. From measured quantitative characteristics we select the 10 that are most associated with good/bad yield (44 good, 132 bad yield), and further the five binary variables in the dataset.

Table 4 shows balanced error rates for the described data and clustering methods. In those examples the use of mixed-data clustering approaches in most cases yielded slightly better results than more common strategies, where either categorical variables are omitted or the data are brought on the same scale. Clustering based on Gower’s similarity coefficient, which is used throughout this paper, performed better than the two mixed-data partitioning algorithms.

**Table 4:** Balanced error rates when clustering three datasets (rows) into two classes of samples using different approaches (columns). The respective smallest BER is highlighted in bold.

	quantitative only	dichotomized	mixed-data clustering		
			latent class	k-prototype	Gower
Cancer treatment response	0.317	0.274	0.274	0.269	**0.262**
Cancer survival	0.356	0.319	**0.314**	0.384	**0.314**
Manufacturing process	0.371	0.375	0.338	0.336	**0.308**

### Exploration of ALL dataset

**Fig 5:**
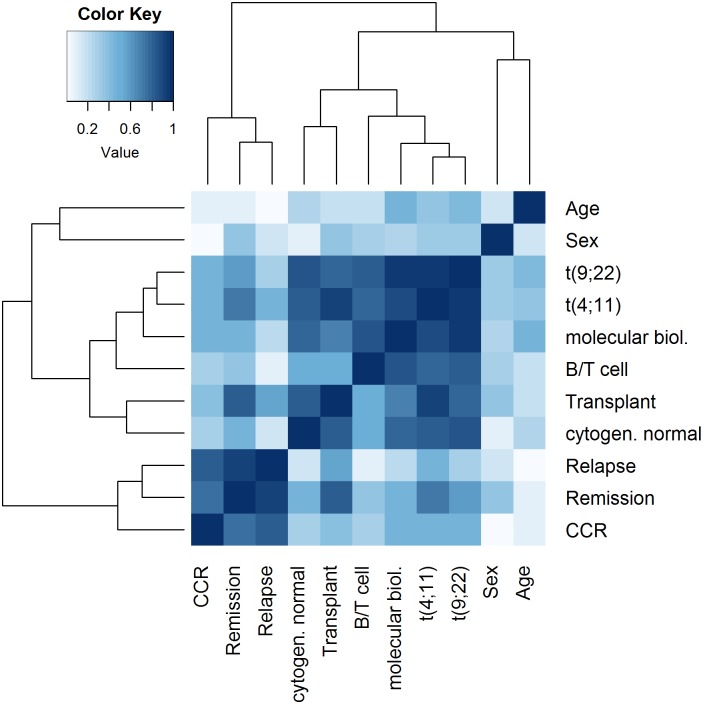
Heatmap of similarities between clinical parameters in ALL. Similarities between variables available in the ALL dataset were calculated by the CluMix-ama approach. Stronger relationships between variables are indicated by shorter distances in the dendrograms and darker blue color in the heatmap.

To give some use case examples for the clustering of mixed-type data and to demonstrate the visualization techniques implemented in our R package *CluMix*, we analyzed a public dataset of 128 patients with acute lymphoblastic leukemia (ALL) [43], which is also available as the R data package *ALL*. The dataset includes clinical information (age, sex, relapse, remission, continuous complete remission for whole follow-up (CCR), transplant status, ALL type (B-ALL/T-ALL)), as well as molecular parameters (translocations t(9;22) and t(4;11), molecular ALL classification), and microarray gene expression measures. First, a similarity matrix for the available clinical and cytogenetic parameters was constructed using the CluMix-ama approach. The similarity matrix was visualized by a heatmap to give an overview over relationships between the different features, see Fig 5. The color intensity indicates the strength of association for each pair of variables. Further, related variables are grouped together by hierarchical clustering. This display is in general useful e.g. in regression analysis, when strongly related predictors shall be identified in order to avoid redundant information or collinearities in the model. Strongest associations are observed between the molecular biology of ALL and the two chromosomal translocations t(9;22) and t(4;11). Presence of translocation t(9;22) (Philadelphia chromosome) is the strongest indicator for the BCR/ABL subtype in ALL. Similarly, translocation t(4;11) defines the ALL-1/AF4 molecular subtype. Bone marrow transplantation is highly indicated in patients with translocation t(4;11), which can also be seen by the corresponding strong association in the heatmap. Further, the type of ALL (B-ALL or T-ALL) is related to the molecular biology, which was expected, since most molecular ALL subtypes are more prominent in B-ALL as compared to T-ALL. The indicator, whether a patient is cytogenetically normal, is also naturally associated with the main chromosomal translocations in ALL. Another block of high similarities comprises indicators about remission following treatment, CCR and relapse. Those follow-up indicators usually can be expected to be interrelated.

**Fig 6:**
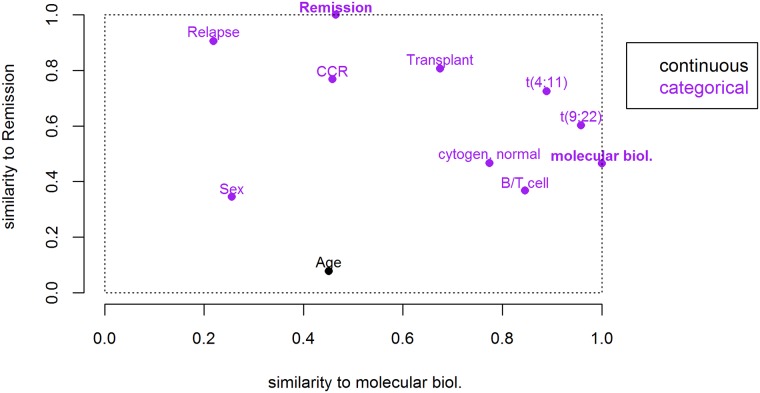
Similarity of each variable with the remission indicator plotted against respective similarities with molecular ALL subtype. From the complete variable similarity matrix (derived by the CluMix-ama approach), the values for two variables of interest, namely remission and molecular subtype, are extracted and shown in a scatter plot, such that each point in the plot illustrates the similarity of a third covariate to both variables of interest. Plotting symbols are replaced by respective variable names. The color indicates numerical (black) and categorical (purple) variables. This kind of illustration may help to identify surrogate, collinear and confounding variables.

Using the same similarity matrix for variables, we further propose a novel type of illustration that might be useful in regression analysis. We assume achievement of remission after treatment as an outcome variable and a the molecular subtypes as potential predictor variable of most interest. From the complete variable similarity matrix the corresponding rows for those two variables are extracted. The similarity values are then shown in a scatter plot, such that each point in the plot illustrates the similarity of the respective third covariate to both the outcome and the predictor, see Fig 6. The outcome and predictor variables themselves are also included in the plot, and obviously take values of 1 at the y- and x-axis, respectively. The x-coordinate of the outcome and the y-coordinate of the predictor correspond to the association between both, and hence give an impression about whether the relationship might be of considerable strength. The positions of all the other variables allow conclusions about their relation to both the outcome and the predictor: The position of points in y-direction gives a first impression about which covariates might have an effect on the outcome. Variables located very close to the outcome variable’s position could potentially be considered as surrogate outcome. As seen from Fig 5, again the strong relation of remission to relapse and CCR become apparent. Features in the bottom right area of the figure are strongly related to the selected predictor variable, but not associated with the outcome. Those would probably not add substantial value to a regression model. If there are variables very close to the predictor variable’s position, this may suggest collinearity. In the example, this seems to be the case for ALL type and cytogenetic features. This makes sense, since the specific translocations define certain molecular subtypes, and almost all patients with T-cell ALL have a NEG subtype. And, last but not least, the figure can help to identify confounding variables, since similarities to both the predictor and the outcome are accessible at a glance. Potential confounders would be located in the top right corner. In this case it seems that transplantations were only conducted in patients with certain molecular subtypes, such that both covariates are confounded. Note that distances between points in the plot do not directly correspond to variable similarities.

**Fig 7:**
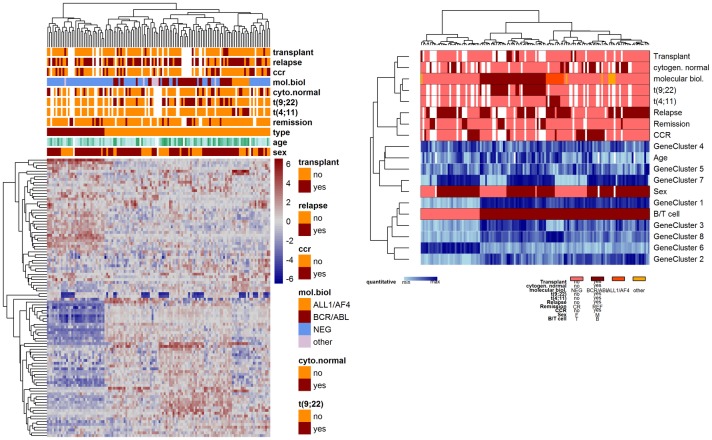
Heatmaps of gene expression and clinical patient data in ALL. The left panel results from a standard approach, where only gene expression data (the 100 most varying genes) are used for clustering. Clinical and cytogenetic information is added on top as color bars. The right panel shows a corresponding novel mixed-data heatmap. The centers of eight k-means gene clusters, together with other clinical parameters, were clustered using the CluMix-ama approach. Patients were clustered using Gower’s distance. Missing values are indicated by white spots.

Next, we used the same dataset to show an example of an unsupervised way to explore global structures among patients and variables, combining both clinical parameters and high-dimensional gene expression data. The microarray data was first reduced by unspecific filtering to the set of the 100 most varying genes. To ease visual inspection, the information within this gene list was further summarized by k-means clustering to eight gene clusters and the cluster centers were then added to the clinical parameters. Patients were clustered using the Gower method. For variables clustering we considered the three approaches described in the Methods section. The certainly most common visualization task to the derived mixed-data distance matrices is the generation of a heatmap, showing structures among variables and patients at the same time. In our mixed-data heatmap, see the right panel of Fig 7, different color schemes are used in order to highlight different types of variables. For numerical features we suggest a blue and for ordinal factors a green color scale. Structures in the data should become visible as areas of light or dark colors, respectively. For non-ordered categorical data we take colors from a red color palette. While for qualitative data usually colors are chosen that do not visually suggest any ordering between categories, one could also argue to make use of the category ordering found in the CluMix-ama approach when calculating similarities between rank ordered and qualitative variables. In this way categories displayed by light/dark red colors can coincide with light/dark colors in variables closest to the categorical variable. Hence, structures in the data become apparent more easily. In Fig 7, patients are observed to group mainly into B-ALL and T-ALL. Within B-ALL patients, further subgroups reflecting different molecular ALL subtypes are visible. The next level in the patient dendrogram hierarchy is dominated by gender. The separation in B-ALL and T-ALL is supported by five of the eight gene clusters. Some of those clusters additionally seem to be associated with the molecular subtypes. For example, cluster 8 apparently contains genes that are mainly up-regulated in the ALL-1/AF4 subtype. Gene cluster 7 appears to be dominated by sex-related genes. The remaining two gene clusters do not show an obvious association with any of the other factors under study. The relations between variables, already discussed above, can be viewed in more detail in this heatmap. For example, the association between continuous complete remission and relapse is obviously negative, and chromosomal translocations t(9;22) and t(4;11) are indicators for the molecular ALL subtypes BCR/ABL and ALL-1/AF4, respectively. To highlight the benefits of the novel mixed-data heatmap, the left panel of Fig 7 shows a “standard” heatmap based merely on gene expression data (here we use again the 100 most varying genes instead of the gene cluster centers). Clinical and cytogenetic parameters are not used for clustering and are just added on top of the image. A grouping of patients into B-ALL and T-ALL is still obvious, which seems to be the main information extractable from gene expression patterns. Further structuring of the patients with respect to molecular subgroups or gender as in the mixed-data heatmap is not apparent here. In general, inspection of the additional parameters is quite cumbersome once there are more than maybe two or three. In contrast, in the mixed-data heatmap they appear as groups of related features, which facilitates to gain a global overview on the dataset.

When using the CluMix-dcor approach or ClustOfVar for the mixed-data heatmap instead of the CluMix-ama approach, we get similar results, see S4 and S5 Figs. However, some differences can be seen, for example using CluMix-dcor gene cluster 5 groups together with translocation t(4;11), whereas with the CluMix-ama approach it is in a group together with age and gene cluster 4. With ClustOfVar only four gene clusters are directly linked to B/T-ALL, whereas gene cluster 8 shows to be closer related to the t(4;11) translocation and the molecular ALL classification. It is usually hard to tell which clustering results “make more sense”, since the “true” grouping of variables is unknown. One can only try to explain which groups of variables are plausible based on the biological background. Here indeed carriers of the t(4;11) translocation seem to have specifically high expression of genes in gene cluster 8. On the other hand, for example the direct link between translocation t(9;22) and molecular subtype BCR/ABL becomes more obvious when clustering with the CluMix-ama and CluMix-dcor approaches as compared to ClustOfVar.

We also produced a heatmap using directly the selected 100 most variable genes instead of gene clusters (see S6 Fig). Patient clustering is then dominated by the separation into B- and T-ALL, whereas further subgroups, i.e. reflecting molecular classification or gender, are not apparent anymore. This is probably due to the fact that the vast majority of genes is related to B- or T-ALL. Introducing variable weights is an option here to give more importance to the minority of clinical and cytogenetic parameters, see S7 Fig for an example. The clustering of variables shows similar groupings as before using gene clusters. Here we can identify directly the genes associated with certain other parameters, without having to go back from clusters to their respective individual members, e.g. B-cell related genes *CD19*, *CD79B* or genes from the *HLA* family (present in gene cluster 1 in Fig 7 and S4 Fig), T-cell related genes *CD3D*, *LCK* and *MAL* (in gene cluster 6), or genes *DDX3Y* and *RPS4Y1* located on chromosome Y and thus related to gender (gene cluster 7).

## Discussion

We described ways how datasets including parameters measured on different scales can be used in cluster analysis—both individual samples and measured variables—while data do not have to be brought on the same scale, which usually would mean loss of information. The main focus of this work lies on i) development and evaluation of new strategies for clustering variables, which we feel is still not a sufficiently well addressed topic in the literature, and ii) integrative visualization, of the preprocessed data itself or of results from statistical analyses. The latter can still be complex in the case of high-dimensional data of possibly different sources.

For clustering samples we used similarity matrices based on Gower’s coefficient of association. In terms of recovering true underlying classes of samples in simulated data (not shown) and real data examples it performs equally well or better than partitioning algorithms, like k-prototype clustering.

For clustering variables i) a new method based on similarity matrices created by combination of association measures (CluMix-ama), ii) a new method based on dissimilarity matrices defined by distance correlation (CluMix-dcor), iii) a hierarchical clustering approach based on PCA for mixed-type data (ClustOfVar), and iv) clustering based on bias-corrected mutual information (BCMI) were evaluated. For approach i) a novel association measure for comparing rank-ordered to qualitative variables based on category reordering was introduced. The different association measures are combined in a way that the resulting distance matrix has Euclidean properties. For approach ii) we derived a new association measure for comparing mixed variables based on the concept of generalized distance correlations. Different from competing measures, this coefficient is 0 if and only if the underlying random variables are independent.

Simulation studies for the new CluMix-ama approach showed that with the proposed measures of association between variables the true underlying correlations of variables w.r.t. their similarities could be captured well. Further simulation studies were conducted to compare the different mixed-data clustering strategies in their ability to recover true underlying structures in the data. In the case of exclusively quantitative data, standard distance measures like the Euclidean distance still would be the first choice. But for mixed-type data such methods are not applicable without data transformation. The simulations showed that in general the use of specific methods for mixed-type variables can be favorable over applying standard approaches to categorized versions of the data—at least for up to around 50% categorical variables in the dataset and for large numbers of samples and/or variables. Approaches i)-iii) for clustering mixed-type variables showed very similar performance. Our two novel methods CluMix-ama and CluMix-dcor perform comparable or, especially in case of larger fractions of non-quantitative variables, better than the existing ClustOfVar approach, and in general they perform better than BCMI.

The two new approaches CluMix-ama and CluMix-dcor provide distance matrices. In contrast to the ClustOfVar approach, this gives the user complete flexibility in terms of which kind of clustering algorithm to apply. Further, similarity matrices are beneficial for visualization purposes. Also BCMI provides a similarity matrix. However, mutual information can be greater than 1, and hence correspondence with correlation values is less clear than for CluMix-ama and CluMix-dcor. If in a dataset linear associations are expected or of most interest, the CluMix-ama approach could be the method of choice, since it was designed to capture correlation-like relationships. For non-linear or even non-monotone relationships, the CluMix-dcor approach might be the best option, since it captures anything differing from independence between variables. If and to which extent such “non-standard” relationships should be reflected in the resulting distance matrix and how the different methods perform in this sense is subject to further research. A further appealing feature of the CluMix-dcor approach is the fact that we calculate distance correlations based on similarities equivalent to the ones used also for clustering samples. Thus, we obtain a unified approach for simultaneous clustering of samples and variables. The proposed methods provide distances where the triangle inequality holds. Nevertheless, it is not clear if certain combinations of data types, e.g. two quantitative variables as compared to one quantitative and one binary variable, systematically yield larger or smaller distances. This issue has to be further investigated and potential corrections have to be developed.

The real data application showed unsupervised illustration of the data. The proposed integrative visualization approach could also be used for supervised settings, e.g., combined visualization of results of supervised feature selection together with further covariates and the outcome of interest (see S8 Fig for an example). Regarding computation time, the CluMix-dcor approach is the fastest for datasets of sizes similar to our simulation studies, and also for larger sets of variables. When increasing sample size, on the other hand, ClustOfVar is more efficient. In our current implementation, the heatmap is limited to a maximum of 200 variables, even though similarity matrices can be calculated for more variables. Hence, dimension reduction is still necessary when working with high-dimensional datasets. Anyway, in most cases feature selection or summarization is necessary to be able to visually distinguish structures in the data.

The use of the presented similarity measures can be extended in several ways. For samples clustering there is also a weighted version of Gower’s distance, which enables to prioritize variables. The weights could be derived, for example, from previous shrinkage regression analysis. Besides explorative analysis, the underlying clustering based on mixed-type data can find other potential applications within the scope of statistical inference. Similarities between samples, assessed using features on different scales, can for example be used for classification tasks. Clustering of variables provides a hierarchy of related features, which can serve as starting point for hierarchical testing approaches.

## Conclusion

The ability to define similarities and to cluster observations and variables of mixed data types is valuable for the analysis and illustration of complex datasets. This work contributes to the methodology of mixed-type variables clustering and emphasizes on integrative visualization strategies.

## Supporting information

S1 Fig Comparison of association measures for different data type combinations. For each comparison 1000 pairs of variables were simulated in the following way: two quantitative variables for 200 samples were simulated to have a Pearson correlation of (from left to right) 0, 0.3, 0.6 or 0.9, as indicated by the red lines. Ordinal, nominal and binary factors were created by categorizing the continuous variables in “perfect agreement”, e.g. by a median cut for the binary factors and quartile cuts for nominal and ordinal factors (resulting in 4 categories). For each combination of variable types, as indicated by the respective titles, three to four different common or new measures of association were applied, where the respective coefficient selected for CluMix-ama in each case is indicated in bold (Pearson = Pearson correlation, Spearman = Spearman correlation, Kendall = Kendall’s tau, GKgamma = Gooman and Kruskal’s gamma, ClustOfVar = similarity measure based on squared canonical correlation as used in ClustOfVar approach, SomersD = Somers’ D, ContCoef = Pearson’s contingency coefficient, CramersV = Cramer’s V, reorderGK / reorderSp = Goodman and Kruskal’s gamma / Spearman correlation applied to “optimal” ordering of categories, see main text). Very similar results were observed when using simulation settings with i) more categories for the nominal variables, ii) some fraction of missing values, iii) smaller sample sizes, iv) unbalanced category sizes for nominal variables (data not shown).
(TIFF)

S2 Fig Misclassification rates from variables clustering of 100 simulated datasets each, varying sample size and number of variables. Datasets were simulated as described in the main article using within-group correlation of 0.5 and 20% of between-group correlations of 0.5 instead of 0. Three approaches for clustering variables were applied: CluMix-ama (top left panel), CluMix-dcor (top right panel), ClustOfVar (bottom left panel) and BCMI (bottom right panel). Simulation settings varied w.r.t. sample size (*n* = 25, 50, 100; panel columns), and numbers of variables (*p* = 50, 100, 200; panel rows). Misclassification rates (MCR) (y-axis) were calculated based on clustering with Euclidean distances for the purely quantitative datasets (orange), with approaches for mixed data for datasets with varying amounts of categorical variables (0%—100%; white to dark blue), and with simple matching coefficient for completely binarized data (green).
(TIFF)

S3 Fig Misclassification rates from variables clustering of 100 simulated datasets each, varying correlation and noise. As S2 Fig, but with fixed numbers of samples and variables of 100, respectively. Simulation settings varied w.r.t. within-group correlations (corr = 0.25, 0.5, 0.75; panel columns), and fraction of between-group correlations with value 0.5 instead of 0 (noise = 0%, 20%, 40%; panel rows).
(TIFF)

S4 Fig Heatmap of gene clusters and other patient data in ALL using CluMix-dcor for clustering variables. The 100 most varying genes were clustered by the k-means method into eight clusters. The respective cluster centers, together with other clinical and cytogenetic parameters, were clustered using the CluMix-dcor approach. Patients were clustered using Gower’s distance. Color codes are explained in the legend below. Missing values are indicated by white spots.
(JPEG)

S5 Fig Heatmap of gene clusters and other patient data in ALL using the ClustOfVar approach for clustering variables. As S4 Fig, but using ClustOfVar instead of CluMix-dcor for clustering variables.
(JPEG)

S6 Fig Heatmap of 100 most variable genes and other patient data in ALL. The 100 most varying genes were clustered together with other clinical and cytogenetic parameters using the ClustOfVar approach. Patients were clustered using Gower’s distance. Color codes are the same as in S4 and S5 Figs.
(JPEG)

S7 Fig Heatmap for ALL data where variable weights were used. As S6 Fig, but clinical and cytogenetic factors were given five times more weight than genes in the calculation of Gower’s distances between samples.
(JPEG)

S8 Fig Heatmap of variables selected for prediction of relapse in ALL. As an example of visualizing results of supervised analyses, a model for predicting relapse in ALL patients was built. Firstly, genes associated with relapse were pre-selected. Secondly, a penalized regression model was built with the 42 previously selected genes with unadjusted p-value < 0.01, together with clinical and cytogenetic parameters. The model resulted in final selection of patient age, whether complete continuous remission had been achieved (CCR), and expression of 14 genes. Those selected features are shown in the heatmap, where the CluMix-ama approach was used for clustering variables. A column color bar indicates the status of the response variable relapse.
(TIF)
