# Supplementary figures and images for: Clustering of samples and variables with mixed-type data
Source: PLoS One. 2017 Nov 28;12(11):e0188274. doi: 10.1371/journal.pone.0188274 (PMC5705083; doi:10.1371/journal.pone.0188274)

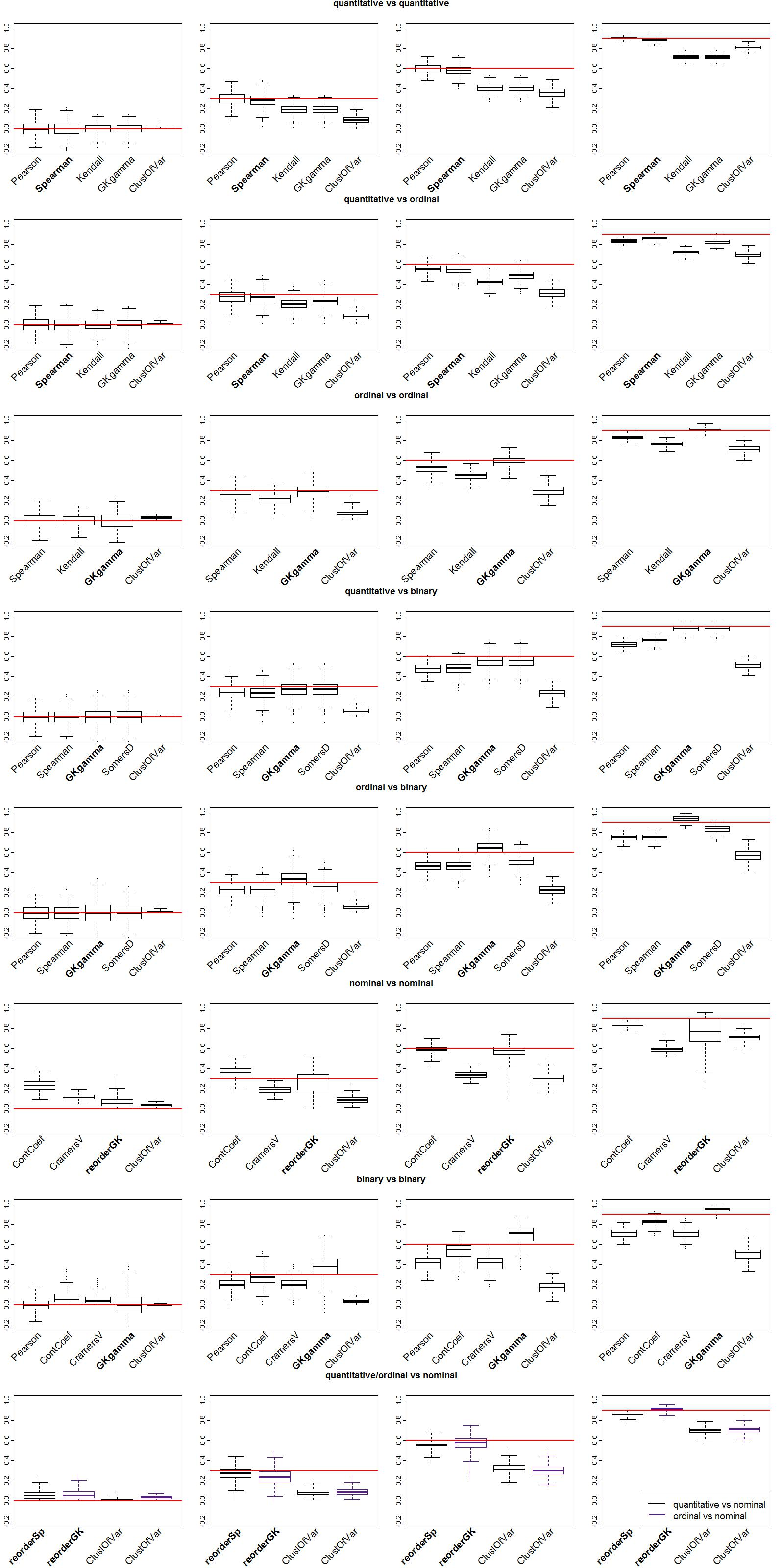

Supplement: S1 Fig — For each comparison 1000 pairs of variables were simulated in the following way: two quantitative variables for 200 samples were simulated to have a Pearson correlation of (from left to right) 0, 0.3, 0.6 or 0.9, as indicated by the red lines. Ordinal, nominal and binary factors were created by categorizing the continuous variables in “perfect agreement”, e.g. by a median cut for the binary factors and quartile cuts for nominal and ordinal factors (resulting in 4 categories). For each combination of variable types, as indicated by the respective titles, three to four different common or new measures of association were applied, where the respective coefficient selected for CluMix-ama in each case is indicated in bold (Pearson = Pearson correlation, Spearman = Spearman correlation, Kendall = Kendall’s tau, GKgamma = Gooman and Kruskal’s gamma, ClustOfVar = similarity measure based on squared canonical correlation as used in ClustOfVar approach, SomersD = Somers’ D, ContCoef = Pearson’s contingency coefficient, CramersV = Cramer’s V, reorderGK / reorderSp = Goodman and Kruskal’s gamma / Spearman correlation applied to “optimal” ordering of categories, see main text). Very similar results were observed when using simulation settings with i) more categories for the nominal variables, ii) some fraction of missing values, iii) smaller sample sizes, iv) unbalanced category sizes for nominal variables (data not shown). (TIFF) [file pone.0188274.s001.TIFF]

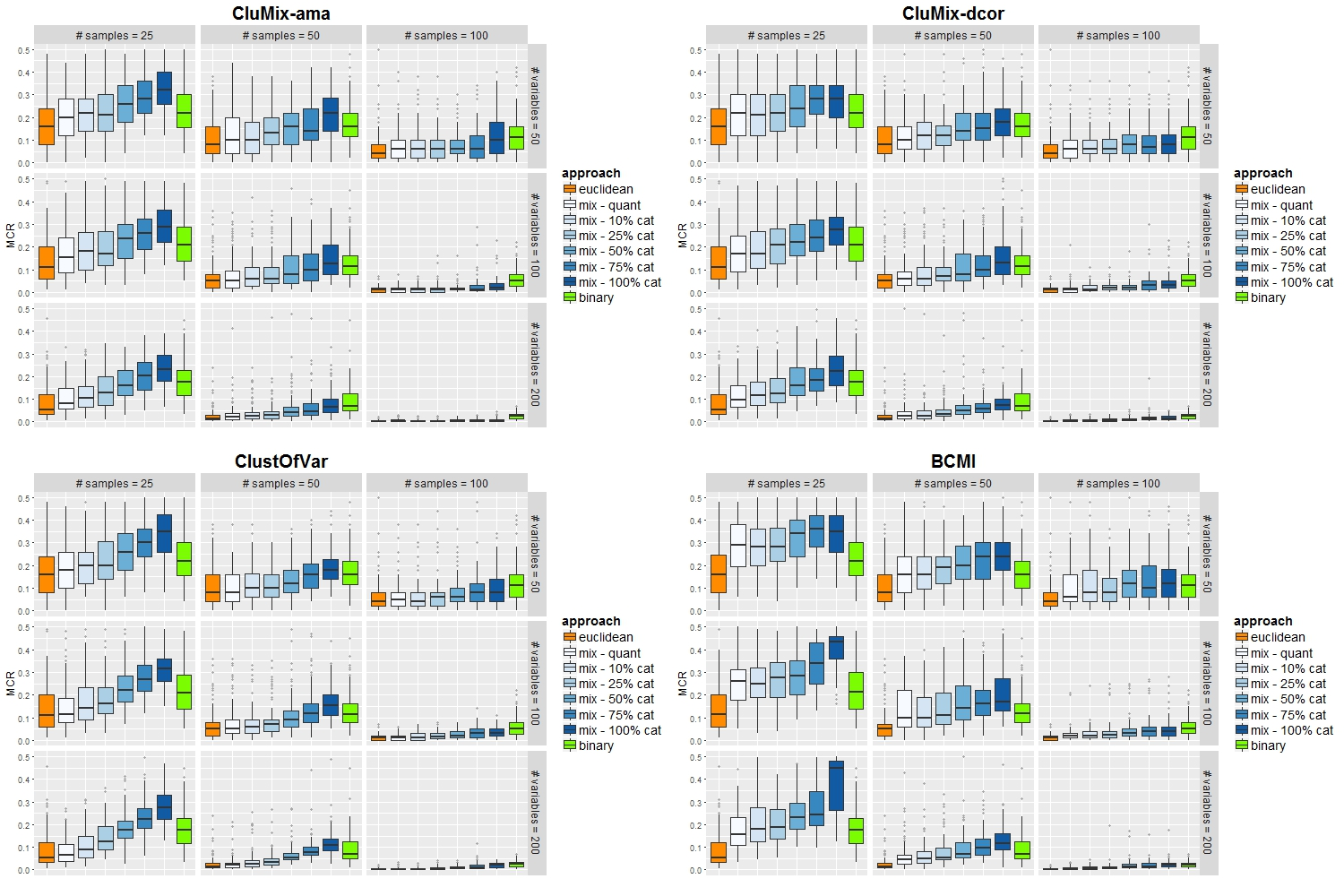

Supplement: S2 Fig — Datasets were simulated as described in the main article using within-group correlation of 0.5 and 20% of between-group correlations of 0.5 instead of 0. Three approaches for clustering variables were applied: CluMix-ama (top left panel), CluMix-dcor (top right panel), ClustOfVar (bottom left panel) and BCMI (bottom right panel). Simulation settings varied w.r.t. sample size (n = 25, 50, 100; panel columns), and numbers of variables (p = 50, 100, 200; panel rows). Misclassification rates (MCR) (y-axis) were calculated based on clustering with Euclidean distances for the purely quantitative datasets (orange), with approaches for mixed data for datasets with varying amounts of categorical variables (0%—100%; white to dark blue), and with simple matching coefficient for completely binarized data (green). (TIFF) [file pone.0188274.s002.TIFF]

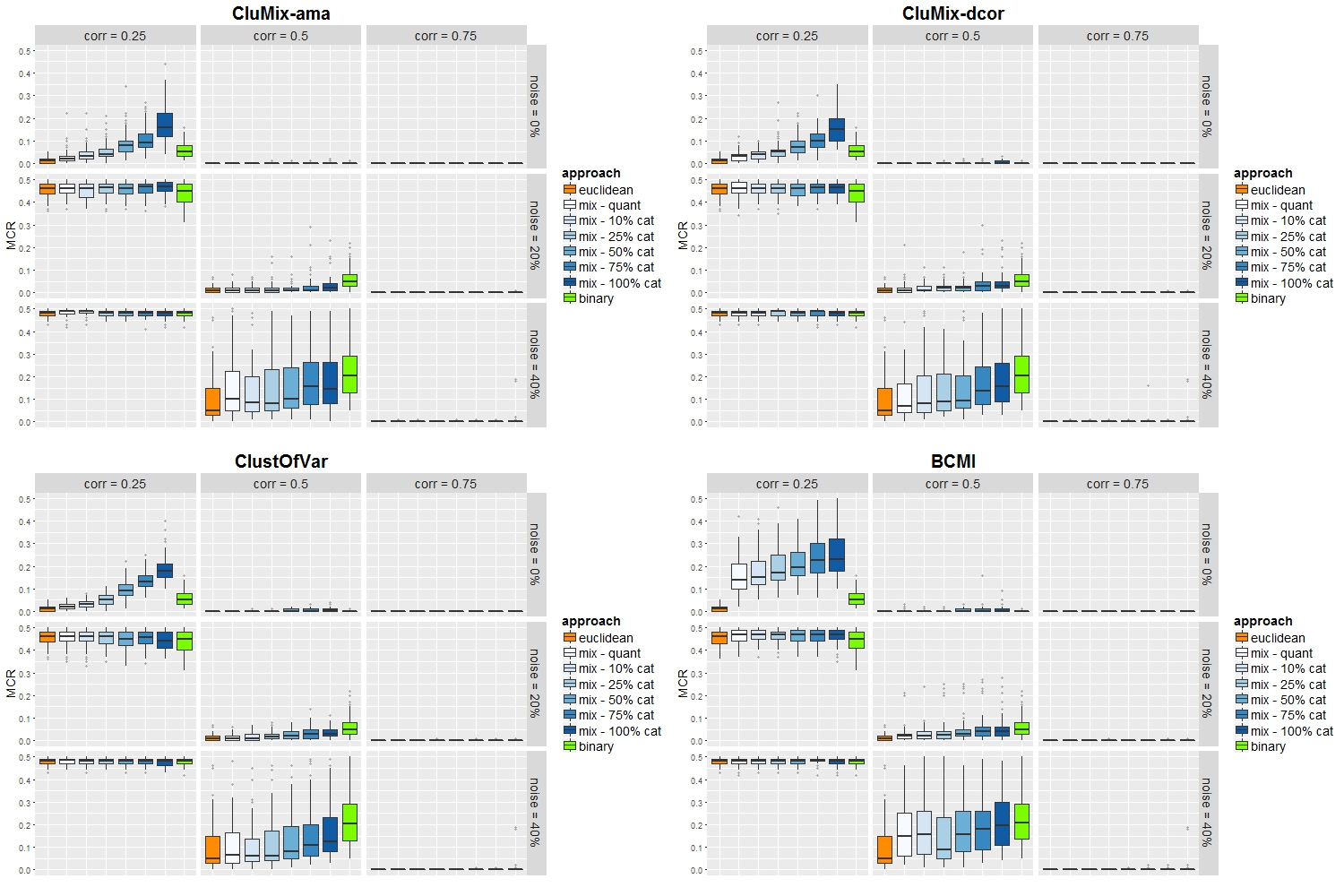

Supplement: S3 Fig — As S2 Fig, but with fixed numbers of samples and variables of 100, respectively. Simulation settings varied w.r.t. within-group correlations (corr = 0.25, 0.5, 0.75; panel columns), and fraction of between-group correlations with value 0.5 instead of 0 (noise = 0%, 20%, 40%; panel rows). (TIFF) [file pone.0188274.s003.TIFF]

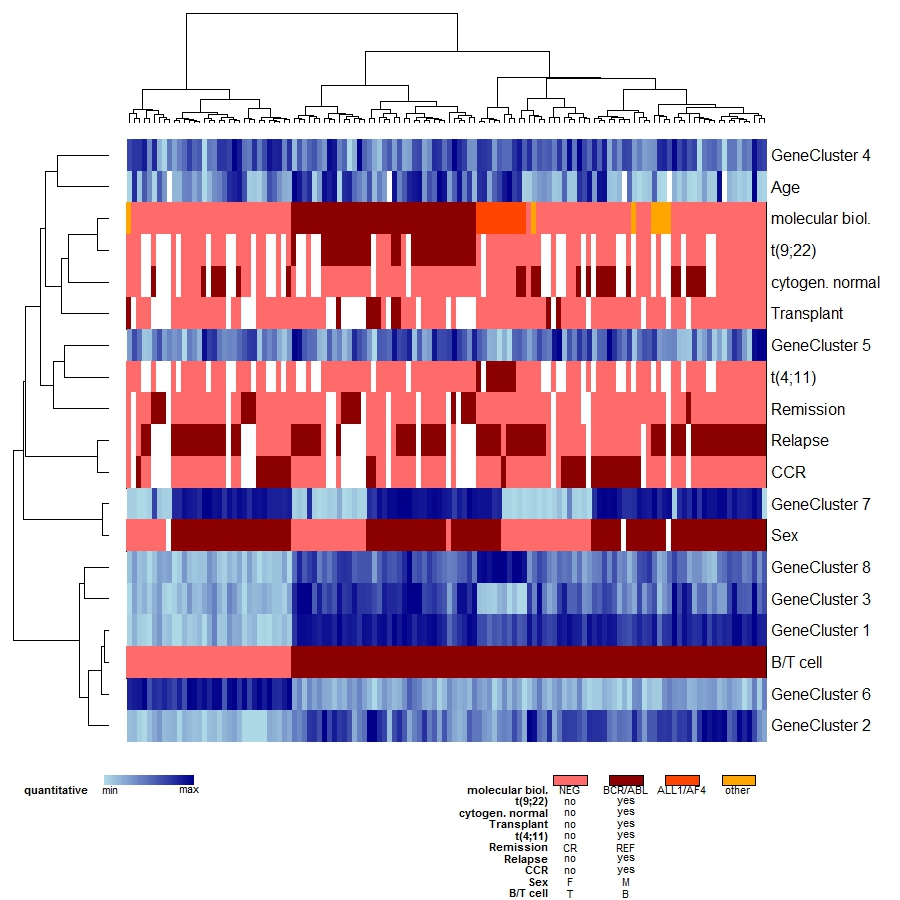

Supplement: S4 Fig — The 100 most varying genes were clustered by the k-means method into eight clusters. The respective cluster centers, together with other clinical and cytogenetic parameters, were clustered using the CluMix-dcor approach. Patients were clustered using Gower’s distance. Color codes are explained in the legend below. Missing values are indicated by white spots. (JPEG) [file pone.0188274.s004.jpeg]

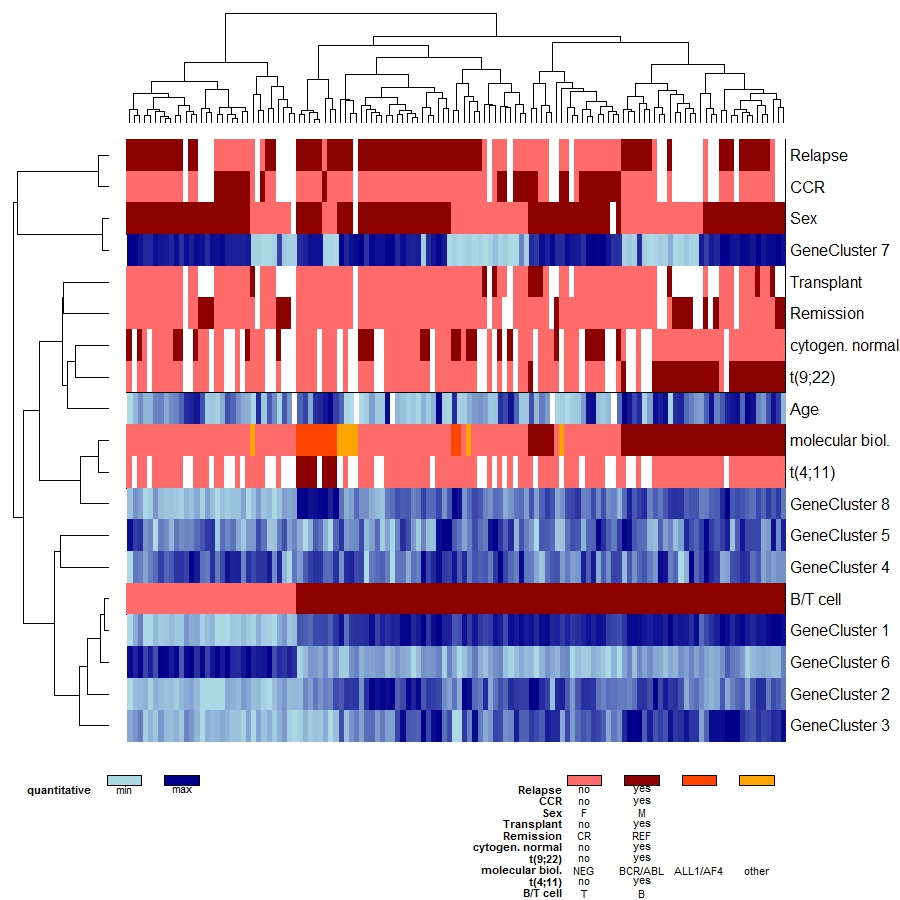

Supplement: S5 Fig — As S4 Fig, but using ClustOfVar instead of CluMix-dcor for clustering variables. (JPEG) [file pone.0188274.s005.jpeg]

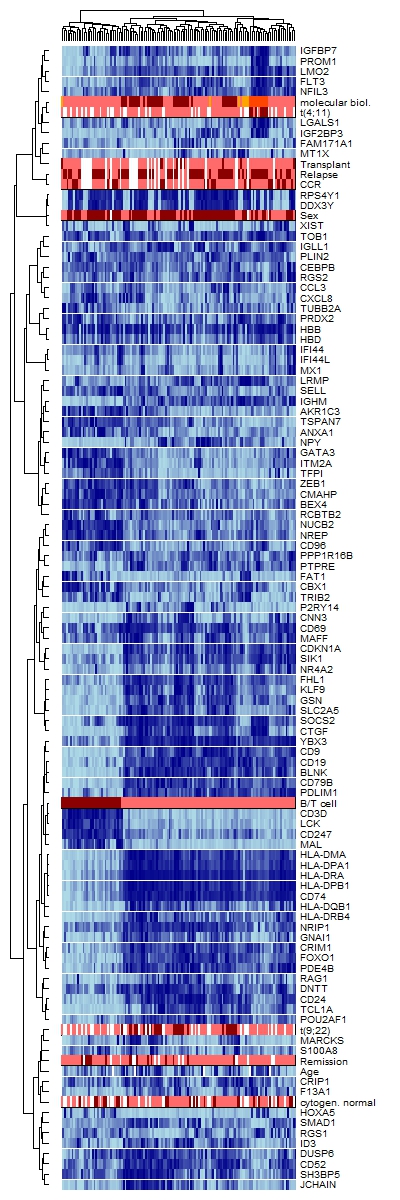

Supplement: S6 Fig — The 100 most varying genes were clustered together with other clinical and cytogenetic parameters using the ClustOfVar approach. Patients were clustered using Gower’s distance. Color codes are the same as in S4 and S5 Figs. (JPEG) [file pone.0188274.s006.jpeg]

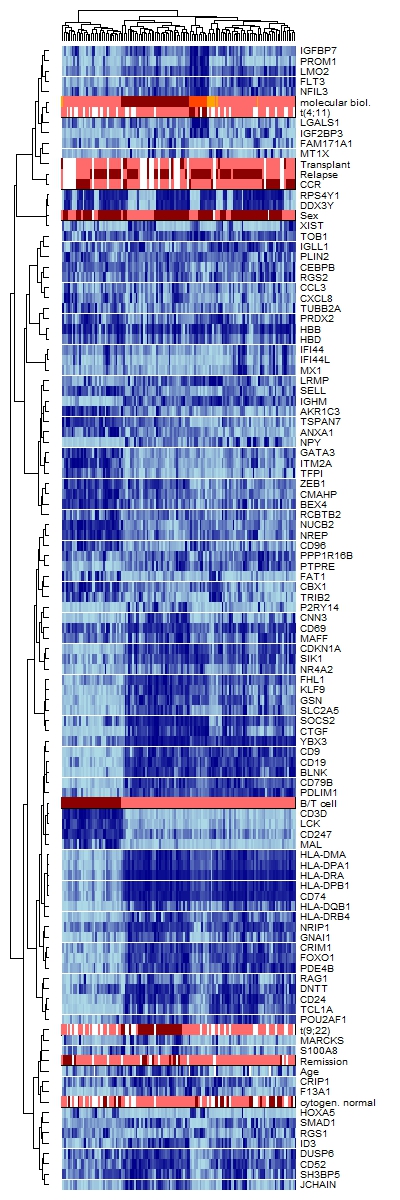

Supplement: S7 Fig — As S6 Fig, but clinical and cytogenetic factors were given five times more weight than genes in the calculation of Gower’s distances between samples. (JPEG) [file pone.0188274.s007.jpeg]

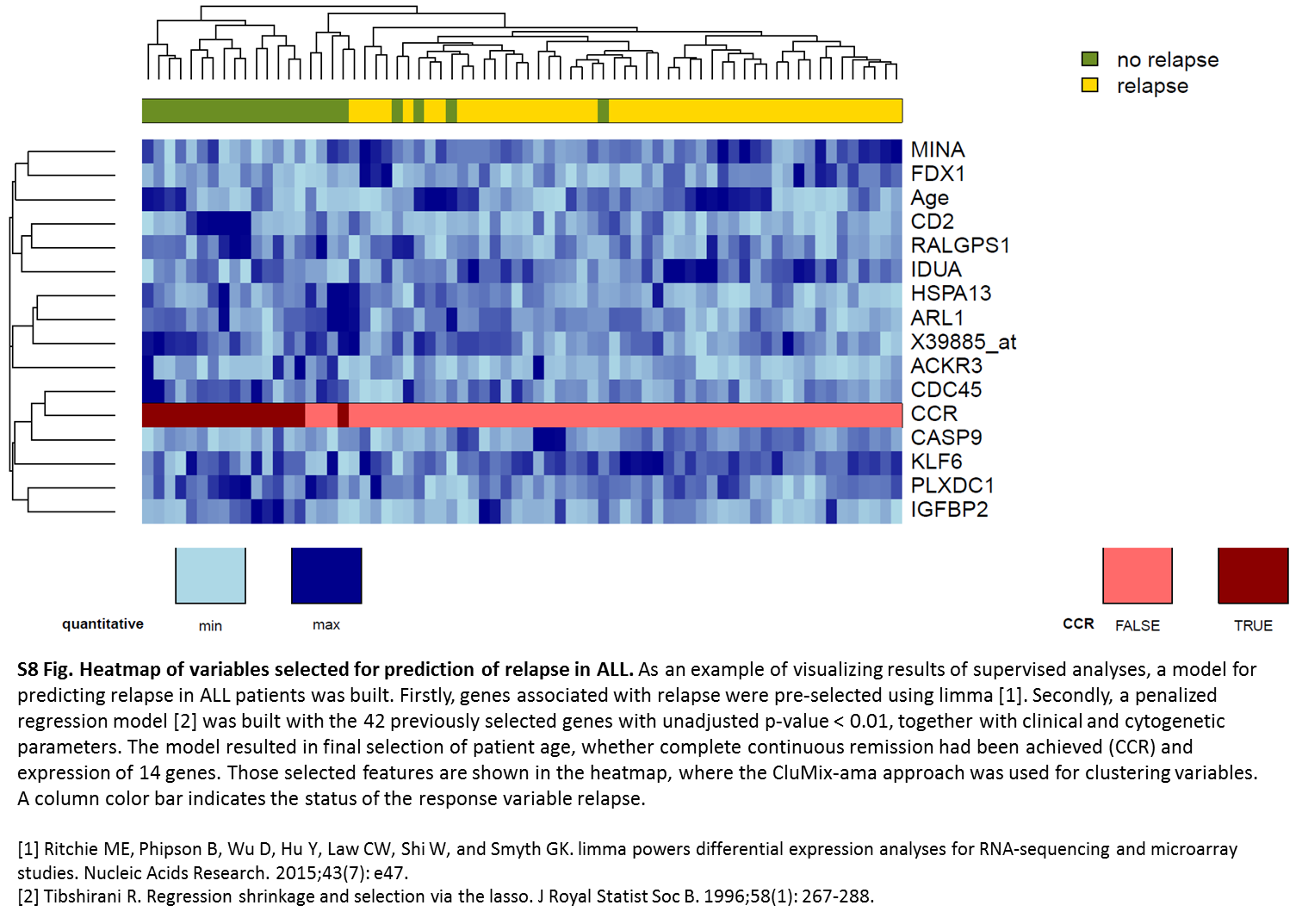

Supplement: S8 Fig — As an example of visualizing results of supervised analyses, a model for predicting relapse in ALL patients was built. Firstly, genes associated with relapse were pre-selected. Secondly, a penalized regression model was built with the 42 previously selected genes with unadjusted p-value < 0.01, together with clinical and cytogenetic parameters. The model resulted in final selection of patient age, whether complete continuous remission had been achieved (CCR), and expression of 14 genes. Those selected features are shown in the heatmap, where the CluMix-ama approach was used for clustering variables. A column color bar indicates the status of the response variable relapse. (TIF) [file pone.0188274.s008.tif]
